# Reverse Mounting in Vultures: A Sexual Tutoring Hypothesis

**DOI:** 10.3390/biology15181610

**Published:** 2026-09-12

**Authors:** Félix Martínez, Guillermo Blanco

**Affiliations:** 1 Escuela Internacional de Doctorado, Universidad Rey Juan Carlos (URJC), C/Tulipán s/n, Móstoles, 28933 Madrid, Spain; femolivas@gmail.com; 2 Department of Evolutionary Ecology, Museo Nacional de Ciencias Naturales (CSIC), C/José Gutiérrez Abascal 2, 28006 Madrid, Spain

**Keywords:** behavioural plasticity, copulatory competency, *Gyps fulvus*, reverse mounting, social mediated learning

## Abstract

Mating performance requires complex motor skills and inter-individual synchronization, yet the developmental pathways of copulatory competency remain poorly understood. Reverse mounting, where females mount males, is integrated into the behavioural toolkits of various monomorphic, long-lived birds, but its adaptive function remains debated. Using a 35-year demographic dataset of a wild griffon vulture (*Gyps fulvus*) population, we tested whether reverse mounting functions as a mechanism of ‘sexual tutoring’ during early reproductive life. Longitudinal observations of individuals marked as nestlings (*n* = 455) and tracked through their reproductive stage revealed that, out of 1498 total observed copulations for them, 1.5% corresponding to 23 cases were reverse copulations involving 9 marked individuals of known exact age. Reverse mounting showed a significant decrease with age, being concentrated in earlier stages with a mean age of 7 years, where 95.7% of these interactions featured an adult female paired with a younger male, and virtually disappearing by 12 years of age with the exception of a single record involving a 25-year-old individual. Subadult females-on-adult males mounting was never observed. While breeding attempts featuring observed reverse copulations showed reduced laying probability, final breeding success was correlated with the individual’s chronological age rather than the behaviour itself. Our findings provide correlational evidence consistent with the hypothesis that reverse mounting may act as a socially mediated learning process. In large-bodied, monomorphic species with high mate fidelity, experienced partners could mitigate asymmetric skills by tutoring youth, potentially accelerating the acquisition of necessary copulatory dexterity.

## Simple Summary

Mating requires complex physical skills that take time to master, but how animals learn these behaviours is often a mystery. In long-lived birds like griffon vultures, females sometimes mount males, a behaviour known as “reverse mounting.” By studying a unique 35-year dataset, we show that this behaviour is not random; our findings suggest it may act as a form of “sexual tutoring.” Younger males may learn essential mating skills through these interactions with experienced adult females. This hypothesized socially mediated learning process helps birds overcome the physical challenges of reproduction, ensuring that they gain the necessary coordination to successfully breed as they transition into adulthood.

## 1. Introduction

Mating performance requires a motor skill that scales with age and experience, a process further enhanced by prior social reinforcement, which reduces misdirected (ectopic) or poorly positioned mounts as a consequence of learning [1,2,3]. Sexually experienced individuals transition more rapidly from appetitive to consummatory behaviours, demonstrating that sexual vigour and coordination are plastic traits mediated through immediate neurobiological rewards [4]. This plasticity is further evidenced by the fact that copulatory behaviour often extends beyond direct fertilization, serving essential social functions like pair-bond maintenance, coordination, and the negotiation of reproductive decisions [5,6].

While the factors driving variation in copulatory repertoires remain poorly understood in birds, morphology, body mass, and agility impose functional constraints on cloacal alignment across avian taxa [6,7,8]. Achieving the necessary balance for successful contact requires specific postural adjustments inherently shaped by these physical limitations. Consequently, the efficacy of mating performance appears to involve a component of learned competency that relies on both individual dexterity and inter-individual synchronization between partners to fulfil both reproductive and social requirements [5,9,10]. Within the diversity of avian copulatory repertoires, reverse mounting, in which the female adopts the male role by mounting her partner during copulation, has been documented in at least 42 bird species across 11 orders [11]. Although its prevalence is likely underestimated due to the difficulty of visually sexing monomorphic species, this behaviour exhibits high variability, ranging from anecdotal occurrences to being an integral component of the mating repertoire [11]. This behaviour has been most frequently documented in species characterized by monogamous mating systems and long-term pair bonds involving biparental care, suggesting it may act as a sexual stimulus to maintain the bond. Several other hypotheses have been proposed to explain its function, including asserting dominance, increasing fertility through ovarian stimulation, acting as a supernormal sexual stimulus for partners with lower receptivity, or playing a role in the mate selection process (reviewed in [11]).

In most studies, the exact age of individuals engaging in complex social behaviours, including reverse mounting, remains unknown [11]. This critical knowledge gap stems from the extreme difficulty of determining precise age and sex through external characteristics alone once subadult or adult stages are reached. This difficulty is magnified in long-lived species with delayed breeding due to the prolonged time elapsed between marking individuals as nestlings to know their exact age and the onset of their reproductive stage. Furthermore, this lifelong longitudinal tracking requires a continuous, long-term monitoring effort, posing a particularly demanding logistical challenge in long-lived, difficult-to-manage species such as vultures. Consequently, this lack of reliable demographic data has severely hindered a comprehensive understanding of rare behavioural phenomena, precluding rigorous testing of evolutionary hypotheses linking age, sex, and individual experience in copulatory behaviour and reproductive performance. Specifically, the absence of detailed demographic information regarding individuals implicated in reverse mounting has impeded the evaluation of hypotheses proposed to explain this behaviour as a learning process for refining copulatory techniques and inter-individual coordination [2,3,4,5]. This behavioural acquisition may be particularly valuable in species characterized by extended lifespans, lifelong pair bonds, and morphological or physical constraints on copulation, such as large body size, reduced agility, or restrictive mating substrates [6,7,8,9].

In this study, the pattern of reverse copulations in the griffon vulture (*Gyps fulvus*) was documented based on a relatively large sample of individuals marked as nestlings and, consequently, of known exact age, followed throughout their lives, especially the early phases of their reproductive stage, over the last 35 years in a large colony. By capturing this multi-decadal continuum from the nestling stage onward, our approach moves beyond the cross-sectional snapshots typical of earlier work, enabling us to directly examine age-specific behavioural enactment. Our primary objectives are to (1) quantify the occurrence and demographic distribution of reverse mounting; (2) evaluate the relationship between age, behavioural patterns, and reproductive success; and (3) propose the novel “sexual tutoring hypothesis” to deepen our understanding of reverse copulations as a developmental mechanism.

This hypothesis posits that reverse mounting manifests as a form of sexual practice or learning during the initial stages of an individual’s reproductive life. Accordingly, such interactions would be expected to function as a mechanism by which older, more experienced partners bridge the pair’s asymmetric skills, leading to an age-dependent dynamic where younger naive individuals may benefit from the guidance of older counterparts (tutors) to achieve behavioural refinement. Specifically, this framework predicts that reverse copulations will be heavily skewed toward the earliest reproductive years, peaking when young, inexperienced individuals first enter the breeding population, and will subsequently decline as age progresses and experience accumulates. Furthermore, this framework predicts a clear directional asymmetry: experienced adult females are expected to proactively mount naive young males rather than the reverse, as a naive male initiating such an act from above would face prohibitive coordination challenges in such a large species. By analyzing known-age individuals within dyads where partner ages can differ, we can explicitly test whether these patterns are driven by the focal bird’s absolute youth or by the specific age asymmetry between the breeding partners. This is conceptually distinct from unguided sexual practice or general social habituation, requiring specific empirical substantiation, which we explore by analyzing age-structured behavioural dyads. Furthermore, this framework also allows for the exploration of whether such departures from “traditional” sexual roles represent a dynamic response to lifespan changes, evaluating their effects on reproductive performance.

## 2. Materials and Methods

### 2.1. Study Area and Colony Monitoring

**Figure 1:**
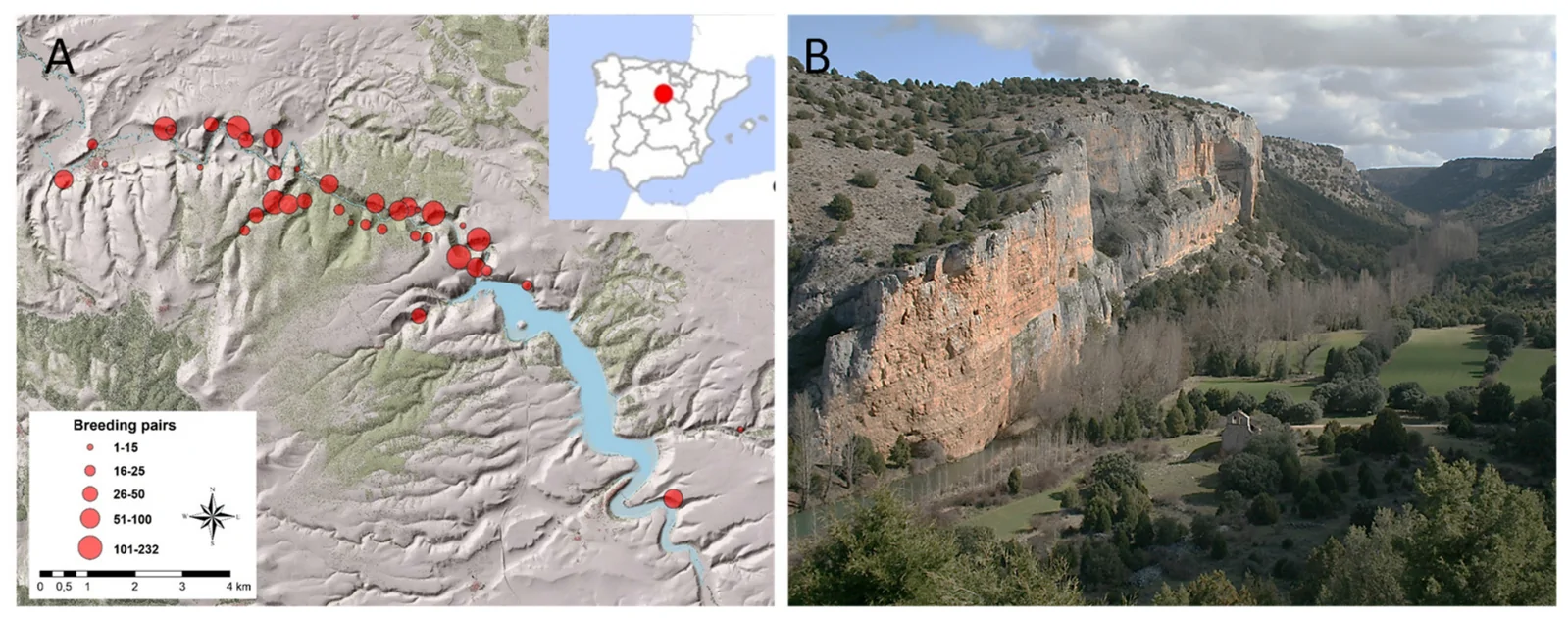
(**A**) Map of the study area showing the spatial distribution of breeding pairs of griffon vultures, with the inset indicating its geographical location in Spain. Red circles represent the number of breeding pairs per cliff, scaled according to the legend (1–15 to 101–232 pairs). (**B**) Representative landscape view of the complex cliff terrain within the study area (credit: F. Martínez). Figure 1A used LiDAR layers. These layers are derived from the Spanish National Plan for Aerial Orthophotography (PNOA-LiDAR), managed by the Instituto Geográfico Nacional (IGN) and the Centro Nacional de Información Geográfica (CNIG), Spain (PNOA-LiDAR, https://pnoa.ign.es/; access date: 10 February 2023). These datasets are freely available for public and academic use in Spain under open reuse licences.

The study was conducted in the Hoces del Río Riaza Natural Park (41°31′ N, 3°36′ W; Segovia, Spain; see Figure 1A for the spatial distribution of nests within the colony, and Figure 1B for an example of the topography and general view of the nesting cliffs), which hosts one of the world’s densest griffon vulture populations [12], reaching 861 breeding pairs in 2025.

Between 1990 and 2025, the reproductive behaviour of most pairs in the colony was monitored through intensive and systematic observations carried out throughout the entire breeding season without causing disturbance. Monitoring consisted of at least five complete annual censuses carried out throughout the entire breeding season (between January and June) following standardized methods [13]. During these censuses, all rocky areas suitable for nesting were surveyed on foot from a distance of 300–500 m opposite the cliffs to avoid disturbing breeding individuals. Nests were observed using binoculars and terrestrial telescopes (20–60×) for as long as necessary—generally between 15 min and 1.5 h, depending on the number of nests present at each site in each season—to determine the status of all present pairs and the presence of marked individuals. Not all pairs could be simultaneously controlled when tracking copulation events (see Section 2.3.). For each breeding pair, we recorded the age of the breeders, the presence of banded individuals, the occurrence of copulation events, incubating individuals, and/or nestlings, while also confirming whether nestlings successfully fledged (breeding success; [12,13]). The exact location of each nest was recorded vertically using GPS, and the cliffs containing the nests were delimited based on their physical continuity and orientation.

### 2.2. Age and Sex Determination

Between 1990 and 2022, vulture nests were accessed by using climbing techniques when the nestlings were at an age of ca. 50–80 days, thus avoiding the risk of premature fledging. The nestlings were banded on-site when the available space at nesting sites was adequate, or alternatively, hoisted using ropes to a safer location where they could be properly processed. Overall, 455 nestlings were individually marked with metal and PVC rings with a unique alphanumeric code for long-distance reading. For these individuals, their exact age can be determined at any time in subsequent years by reading the codes of their PVC rings. Additionally, a total of 177 fully grown individuals were captured using cannon nets and cage traps baited with carrion, and subsequently marked with the same type of PVC rings. The age of individuals captured as fully grown birds was estimated as juveniles (≤2 years old), subadults (3–5 years old), or adults (≥6 years old), based on external characteristics including the colouration of the iris, bill, and plumage, and moult sequence [14,15,16]. Conversely, when dealing with the unbanded mates paired with these marked individuals, a minor limitation or error may arise in the transition between subadult and adult categories when observed at a distance. This arises because the exact age at which each bird acquires final adult plumage can vary by one or two years (at 5 to 6 years of age) between individuals, but the exact age in years becomes completely impossible to determine thereafter [13]. Nevertheless, this limitation does not prevent evaluating at least the developmental or maturity stage of these unbanded individuals, serving as a proxy for their age and reproductive experience.

Sex was determined via molecular DNA analysis from blood samples [17,18] for 304 of the individuals marked as nestlings and for all individuals captured as fully grown birds. For an additional 42 birds marked as nestlings (without DNA samples), sex was confirmed through intensive observations during the days before, during, and immediately after egg-laying, when female-specific reproductive behaviours are clearly distinguishable [19,20].

All field procedures, bird handling, and ringing protocols were conducted under strict adherence to institutional and national animal welfare regulations, approved by the regional government of Castilla y León, Dirección General del Medio Natural, Servicio de Espacios Naturales, and the Hoces del Río Riaza Natural Park, associated with the permits from the Spanish Bird Ringing Centre (Permit Numbers: 530109 to FM and 530115 to GB).

### 2.3. Monitoring of Copulatory Behaviour

Copulatory behaviour was monitored over the study period by focusing on marked individuals of known age. Copulatory behaviour in this species is highly conspicuous due to the emission of loud, persistent grunts that can be heard from a distance, facilitating the location of copulating pairs. When the concentration of breeding pairs was particularly high in certain cliff sectors, not all pairs could be monitored simultaneously; consequently, observations focused on recording copulation events and determining the age and social position of pairs in which at least one individual was individually marked with PVC rings, thereby ensuring knowledge of its exact age. Observations paid particular attention to the copulatory position of marked individuals, recording whether they were in the top or bottom position (mounting or mounted, respectively), as well as the age of both the marked birds and their partners. The age of unmarked partners was estimated via external characteristics as either subadults or adults (see Section 2.2.).

### 2.4. Statistical Methods

To identify the factors determining the probability of the marked individual performing a reverse copulation, we employed a Generalized Linear Mixed Model (GLMM) with a binomial error structure and a logit link function. The analysis was restricted to marked individuals that were individually identified and observed participating in at least one reverse copulation during the long-term study. We acknowledge that this subsetting introduces a methodological boundary, as it excludes other marked individuals only observed engaging in normal copulations. This choice is intentional, allowing us to isolate the specific effect of age and sex on active pairs where reverse copulations were detected—rather than estimating population-wide prevalence—given that it remains unknown whether such events occurred in the remaining pairs. Furthermore, while other marked individuals may have unobserved reverse copulations due to the inherent observational limitations of working with long-lived colonial raptors in complex cliff terrain, our GLMM analyses remain conservative, focusing strictly on confirmed participants while carefully bounding broader inferences.

Observations were coded as 0 for normal copulations and 1 for reverse copulations. The model included the following fixed effects: age of the marked individual in years, which was log-transformed to account for the non-linear relationship across the age range (3 to 25 years); sex of the marked individual (male vs. female); and partner age category, classified based on plumage as either subadult or adult. To test whether the effect of the marked individual’s age on the probability of reverse copulation differed between sexes, we included an interaction term between age and sex in our GLMM. We compared the model with and without the interaction term using a likelihood ratio test (LRT) to determine if the interaction significantly improved model fit. To account for pseudoreplication, the identity of the marked individual was included as a random effect.

To evaluate the influence of reverse copulations on reproductive output, we used the individual ‘breeding attempt’ as the unit of analysis. In this study, a breeding attempt is defined as the entire reproductive cycle of a pair, spanning from the initial courtship and copulatory phase to the final outcome of the attempt, either fledging or failure. This approach was adopted because both normal and reverse copulations can co-occur within a single breeding attempt. The primary response variables defined were the presence or absence of egg laying (binary variable) and the final breeding success (binary variable: failure or production of a fledgling). For the analysis of laying probability, Fisher’s Exact Test was employed because the 100% success rate in laying observed in breeding attempts with normal copulations resulted in a lack of variability within that category; this complete separation of data precluded the use of a GLMM for this specific response. For this analysis, we compared two categories of breeding attempts: (1) those with exclusively male-on-female copulations against (2) those where reverse mounting was observed (including both mixed-behaviour attempts and those with exclusively observed reverse copulations).

To determine the factors influencing final breeding success, a GLMM was fitted with a binomial distribution and a logit link function. The model included the proportion of observed reverse copulations per breeding attempt, the sex of the marked individual, the scaled chronological age of that individual, and the maturity of their partner (categorized as subadult or adult) as fixed effects. The proportion of observed reverse copulations per breeding attempt was determined by dividing the number of reverse mounting events by the total number of copulatory events (regular plus reverse) recorded for that specific pair throughout the entire duration of their breeding attempt. The identity of the marked individual was included as a random effect to control for pseudo-replication and the inherent variability of individuals sampled across different seasons. Scaling the age variable was performed to facilitate model convergence and ensure the comparability of the coefficients.

All analyses were performed in R (version 4.5.3) using the lme4 package, and significance was assessed using Wald z-tests. Model assumptions and fit, including overdispersion and residual distribution, were verified using the DHARMa package.

## 3. Results

### 3.1. Frequency of Reverse Copulations

A total of 1498 copulations were observed involving marked individuals of known exact age and sex, with 1.5% (*n* = 23) identified as reverse copulations. The reverse copulations involved 9 marked individuals (7 males and 2 females), which also performed 85 conventional male-on-female copulations. Overall, reverse copulations accounted for 21.3% (23 out of 108) of the total observed mating events for these specific individuals: 17.4% (16 out of 92) for the individually marked males, and 43.7% (7 out of 16) for the females.

### 3.2. Influence of Age on Reverse Copulations

The mean age of known-age vultures involved in reverse mountings was 7.22 years (SD = 4.74; range = 3–25; mode = 5 and 6; *n* = 23). Most of the reverse copulations (95.7%; 22 out of 23) featured an adult female (>5 years; confirmed by ring code in individually marked vultures or by external characteristics in unmarked ones); in the single remaining case, both members of the pair were subadults (≤5 years). No instances of subadult females mounting adult males were recorded during the study.

**Figure 2:**
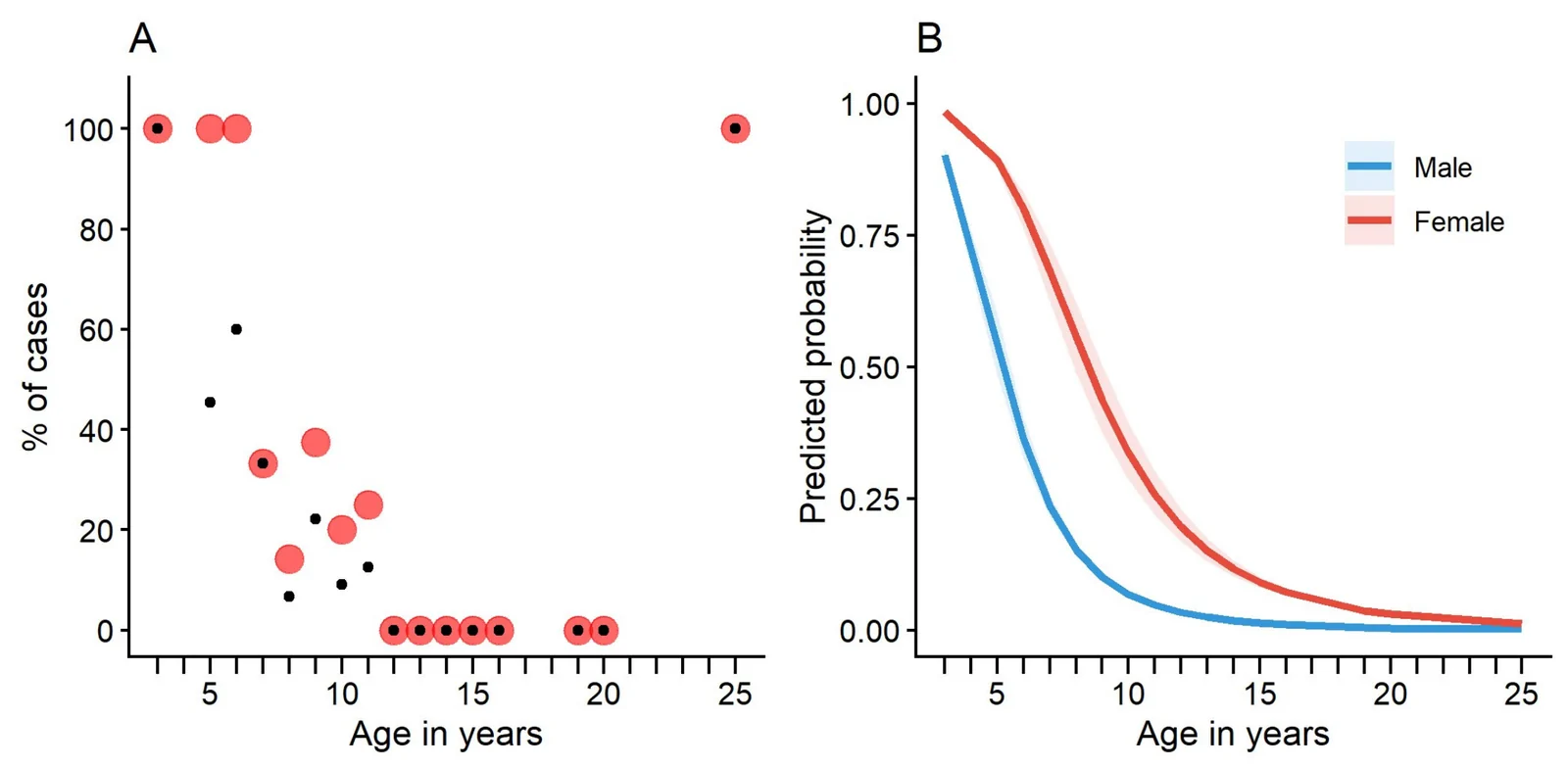
Patterns of reverse copulation by age and sex in marked griffon vultures of known age. (**A**) Percentage of marked individuals that performed at least one reverse copulation at each age (large red circles), and the percentage of total observed copulatory events for these individuals that were reverse copulations (small black dots). Note that at age 3, all observed copulations were reverse mountings (100%), whereas from age 5 onwards, these individuals displayed a mixed repertoire of both reverse and normal copulatory events, explaining the divergence between both metrics. (**B**) Predicted probability of engaging in reverse copulation derived from the GLMM. Solid lines represent predicted values for females (red) and males (blue), with shaded areas indicating the 95% confidence intervals.

The probability of reverse copulation showed a significant decrease as the age of the marked individual increased (Table 1; Figure 2A). This behaviour was most prevalent during early life stages, specifically between 3 and 6 years of age, whereas from 7 years of age onwards, individuals showed a clear transition towards adopting ‘normal’ copulatory roles. By 12 years of age, the behaviour became exceptionally rare until it virtually disappeared, with the exception of a 25-year-old male mated with an adult female in which a reverse copulation was recorded (Figure 2A). There was also a significant effect of the marked individual’s sex (Table 1), with the occurrence of reverse copulations being higher when the marked individual was a female than when it was a male (Figure 2B). In contrast, the partner’s age category (subadult vs. adult) did not show a significant effect on the probability of reverse copulations (Table 1). We also tested for a potential interaction between individual age and sex to determine if the effect of age on the probability of reverse copulations varied between sexes. However, this interaction was not statistically significant, and therefore was not included in the final model.

### 3.3. Influence of Reverse Copulations on Reproductive Output

In breeding attempts with exclusively ‘normal’ observed copulations, all cases (100%, *n* = 33) successfully achieved laying, whereas in attempts with mixed observed copulatory behaviour (normal and reverse), the laying rate fell to 87.5% (*n* = 8), and in those with exclusively reverse observed copulations, the probability of laying dropped to 50% (*n* = 10). Fisher’s Exact Test showed that the occurrence of observed reverse copulations within a breeding attempt significantly reduces the probability of laying compared to attempts where exclusively male-on-female copulations were recorded (*p* = 0.001).

Regarding final reproductive output, attempts with exclusively male-on-female copulations achieved a fledging success of 54.5% (18 out of 33), while in cases of mixed behaviour, no fledgling was produced (0%, 0 out of 8), and in cases where only reverse copulations were observed, the success rate was 20% (2 out of 10).

To account for the potential collinearity between age and copulatory behaviour, we included both variables as simultaneous fixed effects in our GLMM. When evaluating these factors through this model, the scaled age of the marked individual was the only variable with a significant positive effect on the probability of producing a fledgling (Table 1). Conversely, no significant effects were detected for the proportion of reverse copulations or the sex of the individual (Table 1). The maturity (adult vs. subadult) of the partner was excluded from the model because it resulted in complete separation due to the absence of reproductive success among subadult partners, yielding unreliable extreme estimates. The inclusion of individual identity as a random effect yielded a variance of 0.62, which controls for repeated measures within the same subjects and accounts for inherent, unmeasured differences in reproductive performance among individuals.

## 4. Discussion

Our long-term study suggests that copulatory behaviour in the griffon vulture may transcend automated responses, pointing to reverse mounting as a potential plastic strategy associated with age and sex. Although reverse mounting is infrequent at the population level, its occurrence is significantly concentrated in specific pair-bond stages, particularly when inexperienced young males pair with adult females during their first breeding seasons. These findings provide correlational support for the hypothesis that this behaviour may manifest as a form of sexual practice and motor learning during the initial years of reproductive life, when inexperienced individuals transition into the breeding population and establish their first pair-bonds [14]. Because most of these interactions featured experienced adults cooperating with their younger partners, they could potentially facilitate this learning process. This age-dependence is compatible with the concept of sexual tutoring, where a mature partner mitigates asymmetric skills within the pair. This cooperative framework may thus help alleviate the physical and positional awkwardness of younger birds, potentially accelerating their acquisition of the dexterity and inter-individual synchronization required for successful cloacal alignment.

Such a sexual tutoring mechanism manifests when examining how specific adult-young combinations of each sex negotiate these roles. Our data suggest that adult females exhibit a higher probability of reverse mountings on young males, which contrasts with the alternative subadult females-on-adult males mounting, which was never observed. The explanation for this likely lies in the fact that when the female is subadult and the male is adult, the pairing does not result in a reverse copulation but rather a normal one; such asymmetry may help explain the observed difference in frequency of reverse copulations between marked males and females. This pattern is behaviorally parsimonious, as soliciting a reverse copulation from below is presumably far more complex for a young male than for an adult female to proactively initiate the physical act of mounting. Engaging in this reverse interaction may allow the young male to perceive the mechanical demands, balance, and positioning of the copulation from underneath. By being mounted, the young male might experience tactile feedback and learn procedural skills in the habitual male-on-female position. Unlike smaller species, which often possess greater agility during copulation, frequently maintaining balance through wing-flapping or conducting alternative mating postures such as the horizontal copulation [6,21], griffon vultures face severe physical limitations. Their large body size, combined with the often-confined nesting environments (caves and ledges in cliffs) where they copulate, significantly restricts the male’s ability to extend its wings to maintain balance. Consequently, the copulatory act in this species may demand more refined motor coordination and a more complex process of skill acquisition than the mating display of smaller, more agile species in other substrates. Within a monomorphic species, this proactive behaviour by adult tutoring females could conceivably serve as a potent behavioural stimulus and a proposed mechanism for teaching copulatory techniques to an inexperienced partner, expanding tentatively on classic ideas of social selection and mate choice pathways [5,8,10].

Beyond the age-related pattern observed in early life, reverse mounting was observed on a single occasion involving an old male being mounted by an adult female. Rather than necessarily serving a tutoring function, the reappearance of reverse mounting in this case might suggest a plastic compensation for declining physical capabilities, reduced agility, or changing reproductive faculties associated with advanced age. This behaviour potentially ensures the maintenance of the pair-bond even when standard mechanical execution becomes constrained [9,22,23]. While this remains a preliminary interpretation based on a single record in a senescent individual, we suggest it provides an intriguing perspective for considering behavioural plasticity and copulatory flexibility in other avian species.

When translating these behavioural dynamics into direct fitness outcomes, our correlational study shows that breeding attempts including observed reverse copulations can culminate in successful egg-laying and offspring production. However, while egg-laying occurred in all attempts featuring only normal copulations, it decreased to lower rates when both normal and reverse copulations were observed, dropping furthest in attempts where only reverse copulations were observed. Given that field observations capture only a fraction of the total copulatory activity during the fertile period in each breeding season, determining the mechanical effectiveness or sperm transfer efficiency of these reverse events remains challenging [11,23]. Crucially, our analyses suggest that chronological age was correlated with final breeding success. This implies that the lower reproductive output observed in these attempts may be largely a function of the marked individual’s youth and inexperience [12], rather than a direct consequence of reverse copulatory behaviour. Engaging in these interactions during early reproductive life may reflect the broader challenges of youth, representing a transitional learning phase where accumulated age and experience are ultimately the decisive factors for long-term competence [1,2,3,5].

While reverse mounting has frequently been interpreted under the ‘social glue’ hypothesis for pair-bond maintenance, or as a ‘super-normal sexual stimulus’ to elicit interest from less responsive partners [11], our study proposes a novel working hypothesis: a socially assisted learning mechanism. Rather than an aberrant behaviour, reverse mounting appears to be integrated into the behavioural toolkit of this and other vulture species [24]. Given the historical lack of evidence for sperm transfer during reverse mounting [11], we do not view this behaviour as a strictly fertilizatory act; rather, we propose that it primarily functions as a social and behavioural interaction. This practice may have evolved to refine copulatory techniques during early reproductive stages, thus operating as a means to navigate physical constraints, communicate, and calibrate partnerships [5,9,10], and to ultimately maximize subsequent fitness in these mate-faithful, long-lived species with demanding biparental care [25,26,27]. By emerging as a potential pedagogical window in youth primarily driven by adult female proactivity, and a potential compensatory mechanism in old age, reverse mounting illustrates that avian sexual competency can be viewed as a socially mediated process, where cooperation between partners may help overcome biological limitations throughout their reproductive lives [1,2,3,5]. Testing this “sexual tutoring hypothesis” rigorously in other species could uncover new research avenues in the evolution of social and sexual selection.

### Methodological Limitations and Approaches for Future Studies

While our 35-year dataset provides rare longitudinal depth, several important limitations inherent to observational field studies of wild populations must be acknowledged. Because our behavioural observations are correlational, we cannot definitively establish direct causal learning outcomes from reverse mounting alone. However, conducting experimental studies on such processes is exceedingly challenging in wild birds, as manipulation is practically unfeasible and could realistically only be attempted in captivity using smaller, more manageable species. Furthermore, potentially influencing ecological variables, such as the characteristics of nest sites and partners’ health and vigour, may interact with age to affect the probability of promoting and observing reverse copulations. A primary methodological constraint stems from the fact that the partners of our marked individuals were not individually marked, restricting our ability to robustly evaluate dyad-level dynamics and forcing us to rely on external morphological characteristics to estimate partner maturity stage. Of course, obtaining comprehensive individual data for all partners in wild vulture populations is logistically demanding and rarely viable on such a scale. This lack of individual marking for all partners also prevents us from entirely ruling out the potential occurrence of homosexual copulations within the wider dataset, although this possibility was explicitly discarded for focal marked females and those identified by unique plumage whose laying behaviour was confirmed.

Additionally, our field observations capture only a fraction of the total copulatory activity during the fertile period of each breeding season, rendering it difficult to determine the exact mechanical effectiveness or verify sperm transfer in individual events. External factors such as sampling effort or weather covariates were also not formally modelled, meaning potential observer or environmental biases in the detection rate of copulations cannot be fully dismissed. Similarly, there are inherent limitations regarding our analysis of reproductive success. While our evaluations focused specifically on testing whether the presence of reverse copulations and individual age influenced laying probability and breeding success, many other external factors naturally influence reproductive output, such as annual climatic conditions, laying dates, breeder experience, breeding density, inter-pair interference, and predation. However, our study was designed specifically to evaluate whether reverse mounting had a targeted effect on reproductive performance rather than providing an exhaustive analysis of all possible drivers affecting overall reproductive success (e.g., [12,13,15,16]).

Despite these limitations, to our knowledge, this is the first study to analyze reverse copulations in griffon vultures individually marked as nestlings and monitored throughout their lives. Furthermore, our hypothesis introduces a novel research avenue by considering the possibility of a motor learning process that increases with age through the assistance of partners in species that form long-term stable pair bonds. Consequently, this work establishes a starting point for addressing these questions using advanced methodological tools—such as the deployment of automated nest cameras to capture complete copulatory sequences—and motivates future research into sexual behavioural flexibility and pair coordination in long-lived birds.

## 5. Conclusions

This 35-year observational study on a wild population of griffon vultures suggests that reverse mounting is unlikely to be a random or aberrant behaviour, but rather may represent a structured plastic strategy. The findings are consistent with the hypothesis that this behaviour acts as a potential “sexual tutoring” mechanism, in which experienced adult females proactively initiate interactions to help young or subadult males overcome physical and coordination limitations during the early stages of their reproductive lives. Furthermore, although the presence of reverse mountings during a reproductive attempt is immediately associated with a lower probability of egg-laying, final fledging success was correlated primarily with the individual’s chronological age, keeping in mind the correlational nature of our analyses. Taken together, the research highlights the possibility that copulatory competency in long-lived, monogamous birds is a socially mediated process, opening new avenues for understanding social selection and behavioural learning in the evolution of reproductive strategies.

## Figures and Tables

**Table 1:** Generalized Linear Mixed Model (GLMM) outputs examining the predictors of reverse copulation probability and subsequent breeding success.

Response Variable	Predictor Variable	Estimate (β)	SE	z	*p*-Value
Reverse copulation	Intercept	5.04	1.98	2.55	0.011
	Age	−4.02	1.00	−4.01	<0.001
	Sex	1.94	0.68	2.85	0.004
	Partner’s age category	0.86	0.72	1.19	0.232
Breeding success	Intercept	−1.17	0.93	−1.26	0.207
	Age	1.22	0.34	3.63	<0.001
	% of reverse copulations	−1.35	1.04	−1.30	0.194
	Sex	0.32	1.01	0.32	0.749

## Data Availability

The data presented in this study are openly available in Zenodo at https://doi.org/10.5281/zenodo.20555702.

## References

[1] Eberhard W.G. (1985). Sexual Selection and Animal Genitalia.

[2] Woodson J.C. (2002). Including ‘learned sexuality’ in the organization of sexual behavior. Neurosci. Biobehav. Rev..

[3] Domjan M., Gutiérrez G. (2019). The behavior system for sexual learning. Behav. Process..

[4] Sakata J.T., Catalano I., Woolley S.C. (2021). Mechanisms, development, and comparative perspectives on experience-dependent plasticity in social behavior. J. Exp. Zool. A.

[5] Griffith S.C. (2019). Cooperation and coordination in socially monogamous birds: Moving away from a focus on sexual conflict. Front. Ecol. Evol..

[6] Birkhead T.R., Atkin L., Møller A.P. (1987). Copulation behaviour of birds. Behaviour.

[7] Brennan P.L., Orbach D.N. (2020). Copulatory behavior and its relationship to genital morphology. Adv. Stud. Behav..

[8] Wachtmeister C.A. (2001). Display in monogamous pairs: A review of empirical data and evolutionary explanations. Anim. Behav..

[9] Ball G.F., Balthazart J. (2011). Sexual arousal, is it for mammals only?. Horm. Behav..

[10] Roughgarden J. (2012). The social selection alternative to sexual selection. Philos. Trans. R. Soc. B.

[11] Villar D.A. (2024). Reverse mounting in birds. Ethol. Ecol. Evol..

[12] Almaraz P., Martínez F., Morales-Reyes Z., Sánchez-Zapata J.A., Blanco G. (2022). Long-term demographic dynamics of a keystone scavenger disrupted by human-induced shifts in food availability. Ecol. Appl..

[13] Martínez F., Rodríguez R., Blanco G. (1997). Effects of monitoring frequency on estimates of abundance, age distribution, and productivity of colonial Griffon Vultures. J. Field Ornithol..

[14] Blanco G., Martínez F. (1996). Sex difference in breeding age of Griffon Vultures (*Gyps fulvus*). Auk.

[15] López-Rull I., Hornero-Méndez D., Frías O., Blanco G. (2015). Age-related relationships between innate immunity and plasma carotenoids in an obligate avian scavenger. PLoS ONE.

[16] Fargallo J.A., Martínez F., Wakamatsu K., Serrano D., Blanco G. (2018). Sex-dependent expression and fitness consequences of sunlight-derived color phenotypes. Am. Nat..

[17] Wink M., Sauer-Gurth H., Martínez F., Doval G., Blanco G., Hatzofe O. (1998). The use of (GACA)4 to sex Old World Vultures (Aves: Accipitridae). Mol. Ecol..

[18] Gómez-López G., Martínez F., Sanz-Aguilar A., Carrete M., Blanco G. (2023). Nestling sex ratio is unaffected by individual and population traits in the griffon vulture. Curr. Zool..

[19] Fernández J.A. (1977). Comportamiento del buitre leonado (*Gyps f. fulvus*) en nido. Ardeola.

[20] Elosegui I. (1989). Vautour fauve *Gyps fulvus*, Gypaete barbu *Gypaetus barbatus*, Percnoptere d’Egypte *Neophron percnopterus*: Synthèse bibliographique et recherches. Acta Biol. Mont..

[21] Viol L.Y., da Silva Bachetti É., Barçante L., de Azevedo C.S. (2025). A standardised ethogram for the Psittaciformes. Behav. Process..

[22] Wasser D.E., Sherman P.W. (2010). Avian longevities and their interpretation under evolutionary theories of senescence. J. Zool..

[23] Dougherty L.R., Skirrow M.J.A., Jennions M.D., Simmons L.W. (2022). Male alternative reproductive tactics and sperm competition: A meta-analysis. Biol. Rev..

[24] van Overveld T., Sol D., Blanco G., Margalida A., de la Riva M., Donázar J.A. (2022). Vultures as an overlooked model in cognitive ecology. Anim. Cogn..

[25] Sarrazin F., Bagnolini C., Pinna J.L., Danchin E. (1996). Breeding biology during establishment of a reintroduced Griffon Vulture *Gyps fulvus* population. IBIS.

[26] van Overveld T., Blanco G., Moleón M., Margalida A., Sánchez-Zapata J.A., de la Riva M., Donázar J.A. (2020). Integrating vulture social behavior into conservation practice. Condor.

[27] Martínez F., Oltra J., Frías Ó., Gonzalez del Barrio J.L., Pérez-García J.M., Carrete M., Blanco G. (2024). A long-lasting, distant journey of a male griffon vulture informs on the success of differential parental investment. Ecology.

